# Atypical Extraoral Presentation of a Heterotopic Gastrointestinal Cyst on the Face: A Case Report

**DOI:** 10.7759/cureus.64747

**Published:** 2024-07-17

**Authors:** Anita Dhupar, Anupama Mukherjee, Anita E Spadigam, Praveen S Kumar

**Affiliations:** 1 Oral and Maxillofacial Pathology, Microbiology and Forensic Odontology, Goa Dental College and Hospital, Bambolim, IND; 2 Oral and Maxillofacial Surgery, Goa Dental College and Hospital, Bambolim, IND

**Keywords:** hgic, parotid gland, face, cyst, heterotopic gastrointestinal cyst, heterotopia

## Abstract

Heterotopias and choristomas are congenital lesions characterized by the presence of histologically normal tissues at non-physiological anatomic sites. The presence of gastrointestinal tissue in the oral cavity has been recognized as a heterotopic gastrointestinal cyst (HGIC) of the oral cavity. An intestinal heterotopia on the face, in relation to the parotid gland, is extremely rare. Highlighting this possibility is the case of a 42-year-old, non-habitué female with swelling in the parotid region of the face for two years. Clinical examination and radiographic investigations ruled out the possibility of a salivary gland tumor, epidermal inclusion cyst, and enlarged parotid lymph node while confirming the cystic nature of the presenting pathology. Further evaluation was carried out using an excisional biopsy. Histopathological evaluation revealed a cystic space lined by simple columnar epithelium with an abundance of goblet cells. The cystic epithelium was noted to form finger-like projections and crypts. An eosinophilic mucinous content was noted in the cystic space. Using Alcian blue-periodic acid-Schiff (PAS) staining, a distinct Alcian blue positivity of the mucinous material and the goblet cells was noted. This feature confirms the acidic nature of the mucinous content being liberated by the goblet cells. The histopathological features, along with histochemical assessment, were confirmatory for the diagnosis of an HGIC. The patient remains disease-free at the end of a 12-month follow-up. This is the first report of an HGIC at an extraoral site on the face in association with the parotid gland. It highlights the possible presentation of heterotopias in adult patients and warrants clinicopathological vigilance due to its benign nature and late presentation.

## Introduction

Oral cysts with gastric or intestinal epithelium (oral alimentary tract cysts) were recognized and listed in the classification of head and neck cysts as proposed by Shear in 2007 [1]. These cysts represent a unique entity and are considered heterotopias. Heterotopias account for histologically normal tissues presenting at an abnormal site and lack vascular or anatomical connection with the parent organ [2]. These tissues become clinically apparent when proliferation occurs, resulting in a choristoma, or due to the presence of cystic changes and enlargement, as seen in the case of heterotopic cysts [3-5].

Oral alimentary tract cysts, or oral heterotopic gastrointestinal cysts (HGICs) [6,7], are rare entities predominantly reported in pediatric patients with a male predilection [8]. Nearly 60% of HGICs have been reported to involve the tongue, while other sites include the floor of the mouth, larynx, and anterior neck [6,8]. HGICs may clinically present as asymptomatic swellings or pose difficulty in swallowing, speech disturbances, and occasionally a respiratory challenge. Singular cases of HGIC at unusual sites, such as in the submandibular and infratemporal spaces, have been reported [9,10]. Augmenting these varied sites of involvement, we report a novel case of HGIC at an extraoral site, in the parotid region of the face. Distinction of an HGIC from other congenital and neoplastic lesions is a clinical challenge and mandates histopathological evaluation to arrive at a definitive diagnosis. Management strategies include complete excision of the lesion along with long-term follow-up.

## Case presentation

A 42-year-old female with no deleterious or pernicious habits presented to the Department of Oral and Maxillofacial Pathology, Goa Dental College and Hospital, Goa, India, with a chief complaint of swelling on the left cheek. The patient noticed the swelling two years ago after experiencing a momentary, dull aching pain in the parotid region of the face. No aggravating or relieving factors were noted. Over the two years, there was a minimal increase in the size of the swelling with no associated complaints. On detailed anamnesis, there was no history of trauma to the area or any recurrent episodes of pain.

**Figure 1:**
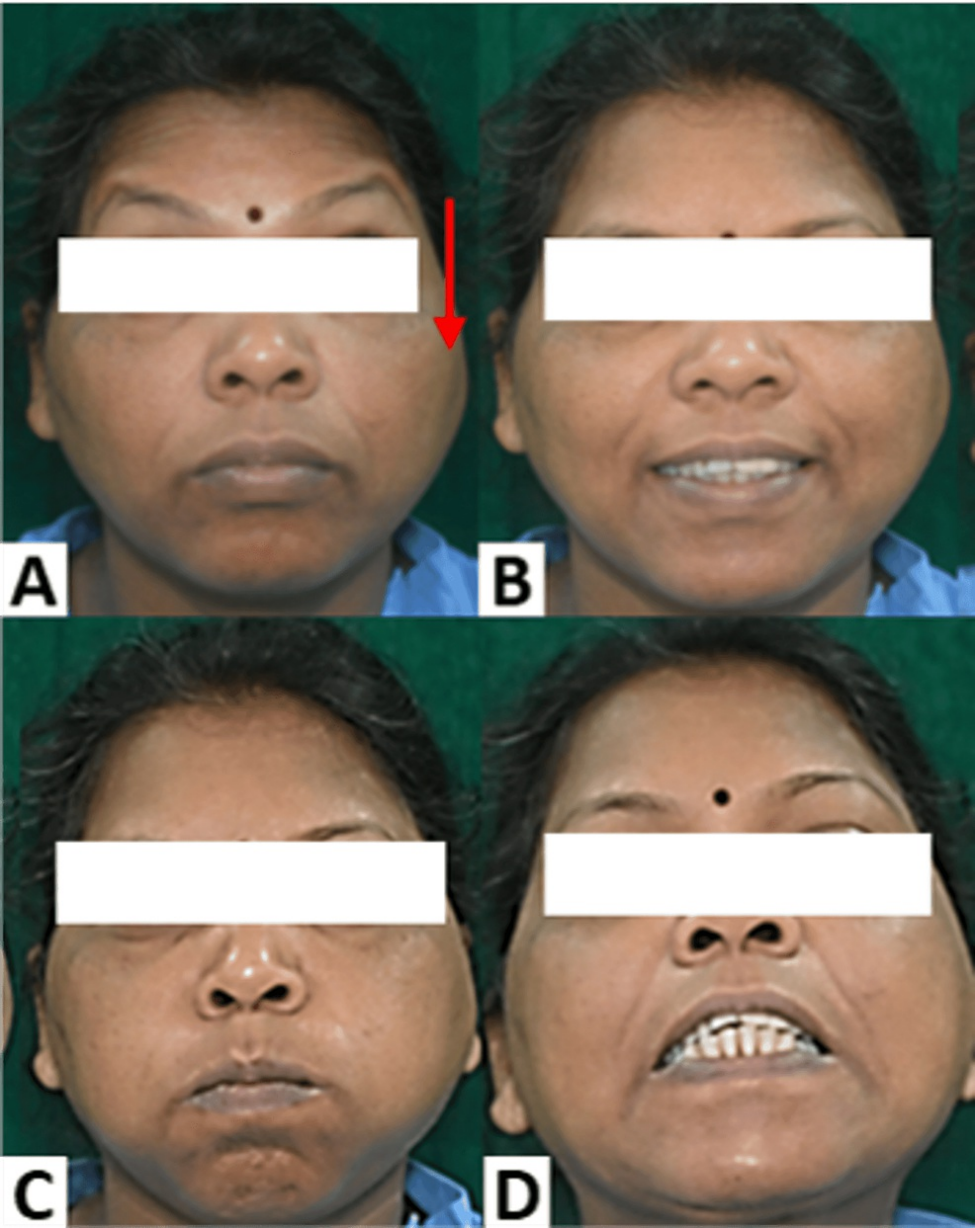
Clinical evaluation. A diffuse swelling is evident on the left parotid region of the face (A, red arrow). Evaluation of the facial nerve for a possible neurological deficit was assessed using the House-Brackmann grading system for facial nerve paralysis and was found to be grade I (normal symmetrical function throughout). All functions such as (A) raising of eyebrows and wrinkling of the forehead, (B) smiling and pulling back the corners of the mouth, (C) pursing of lips and blowing out, and (D) showing of teeth demonstrated no abnormality.

Examination revealed a solitary, diffuse, dome-shaped swelling on the left side of the face in the parotid region about 3 cm anterior to the tragus (Figure 1A). It measured 1 x 0.5 cm and was firm and non-tender. The overlying skin appeared intact with no evidence of sinuses, scars, or fistulas in the peri- and pre-auricular regions. No neurological deficits were noted on the evaluation of the facial nerve (Figure 1). A provisional diagnosis of a salivary gland tumor and parotid lymphadenopathy were considered.

**Figure 2:**
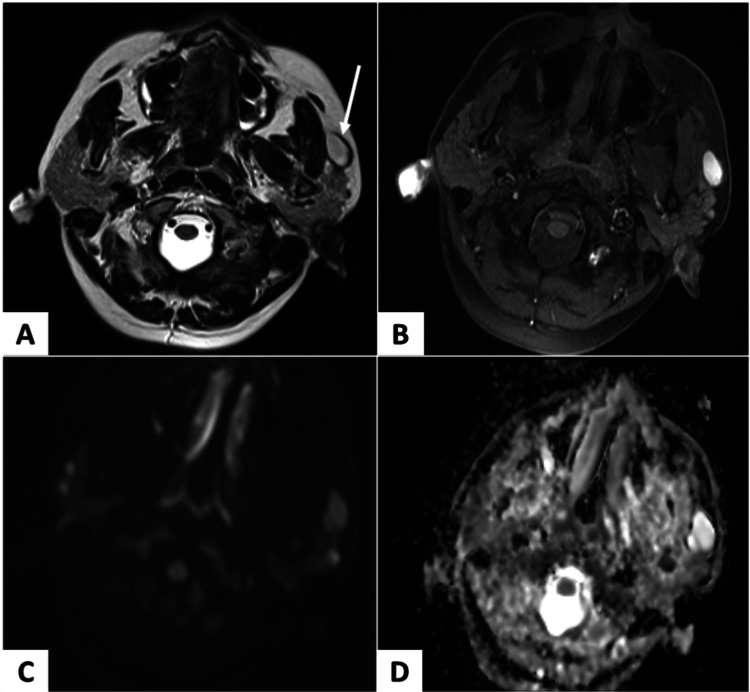
Evaluation using MRI. (A) T2-weighted axial image: A well-defined hyperintense lesion anterior to the left parotid gland (white arrow). (B) T1-weighted fat-suppressed image: Lesion appears hyperintense, most likely due to proteinaceous content within. (C) Diffusion-weighted imaging (DWI) & (D) apparent diffusion coefficient (ADC) images: A high signal was noted on the DWI image with no corresponding low signal on ADC images, suggestive of no restricted diffusion, hence unlikely to be epidermal inclusion cyst.

Ultrasound evaluation of the left parotid revealed a hypoechoic lesion superficial and anterior to the gland. It measured 2.2 x 2 cm with a posterior acoustic enhancement into the intramuscular space. Color Doppler assessment revealed no vascularity in the lesion. These findings were indicative of a cystic lesion. Preoperative MRI on T2-weighted axial images showed a well-defined hyperintense lesion anterior to the left parotid gland (Figure 2A, white arrow). High signal on diffusion-weighted imaging (DWI) with no corresponding low signal on apparent diffusion coefficient (ADC) images were suggestive of no restricted diffusion (Figures 2C, 2D), hence ruling out the possibility of a salivary gland tumor and an epidermal inclusion cyst.

**Figure 3:**
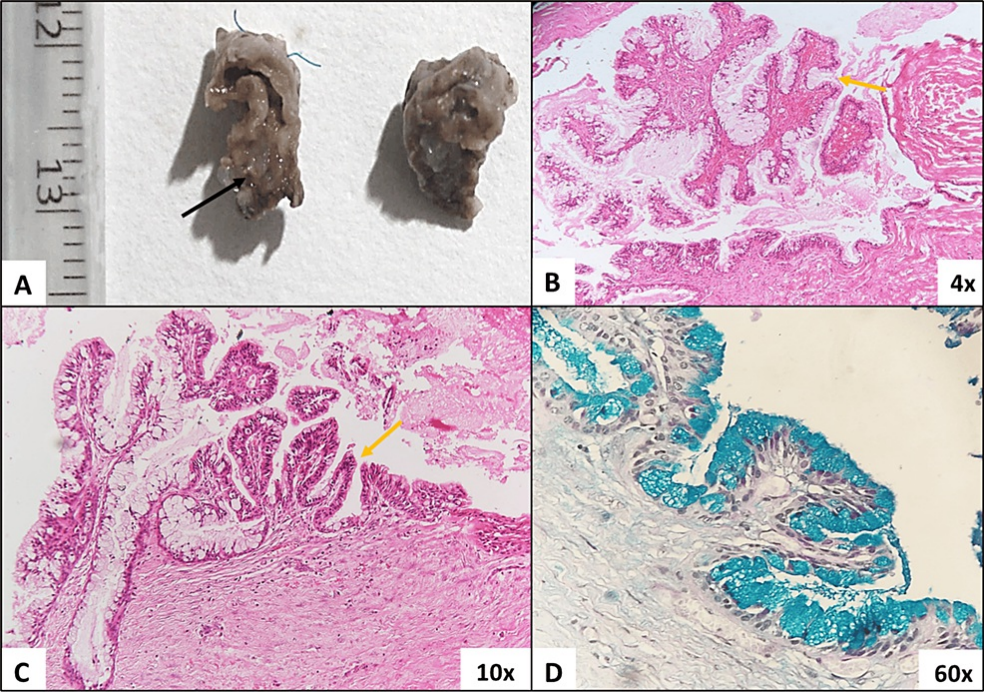
Gross and microscopic evaluation of excisional specimen. (A) Gross evaluation of excisional specimen. The specimen was bisected and showed a cystic space with mucinous material (black arrow). (B) Hematoxylin and eosin-stained section (4x) shows cystic epithelium overlying a mature fibrous connective tissue stroma. The epithelium is proliferating in a finger-like pattern with connective tissue cores. Eosinophilic mucinous content is evident in the cystic space. (C) Hematoxylin and eosin-stained sections (10x) showed cystic epithelium with areas of crypt formation (yellow arrow) comprising of simple columnar epithelium with an abundance of goblet cells. (D) Alcian blue-PAS-stained sections (60x) showed a negativity for PAS and a diffuse positivity for Alcian blue amongst all goblet cells confirming the acidic nature of the mucinous content. PAS: periodic acid-Schiff.

Hematological investigations included a complete hemogram, which was within normal limits. An excisional biopsy was carried out, and the specimen was submitted for histopathological assessment. A bilobed specimen was received and bisected. The cut surface revealed a cystic cavity with a corrugated cystic lining and mucinous content (Figure 3A, black arrow).

Histopathological evaluation revealed a lumen lined by cystic epithelium overlying a moderately cellular connective tissue stroma. The cystic epithelium was seen to be proliferating as finger-like projections resembling villi along a few invaginated areas in the epithelium resembling intestinal crypts (Figures 3B, 3C, yellow arrow). The cystic epithelium comprised simple columnar epithelium with abundant goblet cells (Figures 3B, 3C). The content of the goblet cells was further evaluated using Alcian blue-periodic acid-Schiff (PAS) staining. A significant Alcian blue positivity indicated the acidic nature of the mucinous content (Figure 3D). The underlying stroma was mature fibrous tissue, comprising fibroblasts and collagen fibers. A muscularis layer was not observed, and aggregates of normal-appearing serous acini were noted in the deeper stroma.

These features of the cystic lining, along with the histochemical evaluation, ruled out a dermoid, epidermoid, or benign lymphoepithelial cyst. Each of these lesions demonstrates distinct histopathological features, which were not observed in this case. In this case, the cystic epithelium showed a resemblance to the intestinal epithelium due to the villi and crypt-like pattern of proliferation, the abundance of goblet cells, and the production of acidic mucin. These features confirm the diagnosis of an HGIC with a predominance of intestinal epithelium. The patient has been on a quarterly follow-up for 12 months and remains asymptomatic.

## Discussion

HGIC is a rare congenital anomaly, first recognized by Foderl in 1895 [11]. A total of 68 cases of HGIC involving various sites in the head and neck have been reported, using various terms such as oral choristoma cyst, heterotopic gastric mucosa, and heterotopic intestinal cyst [12]. HGICs commonly occur in the pediatric age group, although 10 of the 68 cases reported in the head and neck have presented in adults [12]. A prevalence of 2.6 times has been noted in males compared to females, with the tongue being the most commonly involved site. Other rarer sites demonstrating HGICs include the hard palate, submandibular gland, and infra-temporal fossa [7-9]. HGICs demonstrate a combination of histologically normal epithelia, such as purely gastric epithelium (29%), gastric along with respiratory epithelium (23%), and intestinal epithelium (21%), and rarely demonstrate intestinal epithelium only (4%) [13]. A rare coalition of HGIC with a dermoid cyst has been reported by Narwal et al. [10].

The present case is an unconventional manifestation of HGIC as it was observed in an adult female at an extraoral site associated with the parotid gland. The clinical presentation mimicked a possible salivary gland tumor. Detailed examination and imaging emerged as essential tools to differentiate a malignancy from a benign cystic lesion. A detailed histopathological evaluation of the cystic lining, cyst wall, and contents of the cystic space helped rule out a dermoid, epidermoid, and benign lymphoepithelial cyst (Table 1) while recognizing the unique histopathological features indicative of an HGIC.

**Table 1:** Histopathological features to distinguish various cysts that may occur in the parotid region in comparison with a heterotopic gastrointestinal cyst (HGIC).

	Dermoid cyst [14,15]	Epidermoid cyst [16]	Benign lymphoepithelial cyst [17]	HGIC [10-12]
Cystic epithelium	Stratified squamous epithelium	Stratified squamous ortho-keratinized epithelium	Ciliated columnar epithelium/cuboidal/flat squamous cells in various areas of the cyst	Presence of gastric/intestinal/respiratory epithelium in combination or independently
Cyst wall	Mature skin appendages (hair follicles and sebaceous glands)	Does not contain eccrine glands, sebaceous glands, or hair follicles	Dense polymorphous lymphoid tissue with germinal centers and sinusoidal spaces	May show the presence of a smooth muscle layer (muscularis layer)
Content of cystic space	Keratin with or without hair shafts opening into the lumen	Abundance of keratin	Keratinaceous/mucinous material with or without the presence of lymphocytes	Mucinous content

Histochemistry proved to be a simple and effective tool to identify the nature of cellular and cystic content. The presence of only intestinal epithelium further categorizes this case as a rare type of HGIC. The absence of a smooth muscle layer, as noted in this case, has also been reported by Bains et al. [12]. Management of HGICs entails surgical excision along with regular follow-up for the rare event of recurrence. Numerous theories have been proposed to explain the pathogenesis of HGICs. Most authors focus on the entrapment of endodermal cells into the tongue and oral anlage during the rupture of the buccopharyngeal membrane. While this could explain the occurrence of intraoral HGICs, the same cannot be extrapolated to explain extraoral presentations as reported by Narwal et al., Kwon et al., and the present case [5,10,11]. As postulated by Woolgar et al. [18], HGIC associated with salivary glands could result from the differentiation of salivary gland tissue into gastrointestinal epithelia, triggered in an existing developmental anomaly such as a salivary gland cyst [11,18]. Conversely, Hanke et al. reported the occurrence of heterotopic salivary gland tissue (HSGT) at various sites in the gastrointestinal tract, postulating this phenomenon as a metaplastic change [19]. This bidirectional appearance of gastric and salivary gland tissues as heterotopias in the respective organ systems needs further exploration and validation.

## Conclusions

There remains substantial ambiguity regarding what initiates and drives the presence of differentiated tissues of endodermal origin in otherwise ectodermally derived structures. It has emerged that HGICs are not exclusive to the pediatric age group and can clinically mimic various benign and malignant lesions. While the present case accounts for the first HGIC reported in the parotid region on the face, the exclusive presentation of intestinal epithelium reinforces its uniqueness and rarity. Clinicians must be aware and vigilant of the possibility of encountering rare anomalies, such as HGICs, at various sites of the head and neck, in addition to conventional sites that have been previously reported.
